# A meta-analysis of randomized controlled trials on atenolol’s impact on postoperative atrial fibrillation in adults undergoing cardiac surgery

**DOI:** 10.1097/MD.0000000000050029

**Published:** 2026-07-31

**Authors:** Jin-He Deng, Fan-Rong He, Yun-Tai Yao

**Affiliations:** a Department of Anesthesiology, The Second Affiliated Hospital of Guangzhou University of Chinese Medicine, Guangzhou, China; b Department of Obstetrics and Gynecology, The Air Force Hospital of Southern Theater Command, Guangzhou, China; c Department of Anesthesiology, Fuwai Hospital, National Center for Cardiovascular Diseases, Peking Union Medical College and Chinese Academy of Medical Sciences, Beijing, China; d Evidence in Cardiovascular Anesthesia (EICA) Group, Beijing, China; e Center of Outcomes Research, Department of Anesthesiology, Critical Care and Pain Medicine, University of Texas, Houston, TX; f Outcomes Research Consortium, Houston, TX.

**Keywords:** atenolol, cardiac surgery, postoperative atrial fibrillation

## Abstract

Prior research has conducted a limited number of studies on the efficacy of atenolol in preventing atrial fibrillation following cardiac surgery (CS). Consequently, a comprehensive evaluation and meta-analysis were undertaken to assess the effectiveness and safety of atenolol in patients undergoing CS for the prevention of postoperative atrial fibrillation (POAF). A meta-analysis of randomized controlled trials was performed. Searches were conducted across multiple databases up to December 1, 2024. The primary focus was the incidence of POAF. Risk ratios (RRs) for treatment effects on dichotomous variables were calculated. The data analysis encompassed 6 randomized controlled trials involving a total of 870 patients. The meta-analysis revealed that atenolol significantly reduces the incidence of POAF in adult patients undergoing CS (RR, 0.55; 95% confidence interval [CI]: 0.32–0.93; *P* = .03) with moderate heterogeneity (*I^2^* = 57%; *P* = .10). Atenolol did not demonstrate superiority over sotalol in reducing POAF (RR, 2.39; 95% CI: 1.41–4.04; *P* = .001) with moderate heterogeneity (*I^2^* = 40%; *P* = .19). Furthermore, no significant difference was observed between atenolol and the control group (comprising propafenone, metoprolol, nebivolol, and digoxin) in the prevention of POAF (RR, 1.28; 95% CI: 0.76–2.16; *P* = .35) with moderate heterogeneity (*I^2^* = 43%; *P* = .15). Recent studies suggest that atenolol could be a safe and effective intervention for the prevention of POAF in adult patients undergoing CS.

## 1. Introduction

Postoperative atrial fibrillation (POAF) is a frequent arrhythmia, affecting 15% to 40% of patients after coronary artery bypass graft (CABG) surgery.^[1–4]^ A 2018 analysis of the EXCEL trial showed perioperative atrial fibrillation (AF) in 18% of CABG patients and 0.1% of percutaneous coronary intervention patients.^[5]^ AF occurs in 37% to 50% of valve surgery patients and up to 60% in those undergoing both valve replacement and CABG.^[1,6,7]^ Most atrial arrhythmias appear within the first few days postsurgery, with a study showing initial AF episodes by day 2 and recurrent episodes by day 3, affecting 43% of patients.^[1,2,8,9]^ POAF in cardiac surgery (CS) patients can lead to stroke, death, and longer hospital stays.^[2,6,10,11]^ Effective interventions are essential, with current guidelines recommending beta-blockers to prevent and manage tachyarrhythmias.^[12]^ However, no specific beta-blocker is favored due to insufficient evidence. Overall, POAF is a major concern, and beta-blockers are crucial in reducing its risk and complications, making their routine use perioperative important.

Accurate preoperative risk stratification for POAF remains critical for targeted prophylaxis. Established predictors of POAF encompass both electrocardiographic and inflammatory biomarkers. Specifically, the morphology–voltage–P-wave duration electrocardiogram risk score has demonstrated prognostic value in identifying patients at elevated risk, with differential performance observed between single and bilateral internal thoracic artery grafting strategies.^[13]^ In addition, inflammatory biomarkers such as the monocyte-to-high-density lipoprotein ratio have emerged as significant predictors of POAF following aortocoronary bypass graft surgery, reflecting the role of systemic inflammation in the pathogenesis of postoperative arrhythmias.^[14]^ Identification of high-risk patients preoperatively enables more selective initiation of prophylactic therapies, such as atenolol, and may ultimately improve clinical outcomes through a personalized approach to POAF prevention.

Atenolol, a selective β1-adrenergic receptor blocker, is frequently employed in clinical settings to treat hypertension and supraventricular tachycardia. Evidence suggests that atenolol significantly decreases the occurrence of POAF in patients undergoing CS compared with a placebo.^[15–17]^ Despite these findings, research on atenolol’s effectiveness in preventing AF post-CS remains limited. Additional rigorously designed clinical trials are necessary to thoroughly assess the role of atenolol in the effective management and prevention of these conditions.

This study evaluates atenolol’s effectiveness and safety in preventing POAF in CS patients, aiming to strengthen evidence-based decisions and improve clinical outcomes while guiding future research.

## 2. Methods

The meta-analysis protocol, registered with the Prospective Register of Systematic Reviews (ID: CRD42023391757), follows Preferred Reporting Items for Systematic Reviews and Meta-Analyses guidelines to ensure high research quality and transparency.^[18]^

### 2.1. Search strategy

We conducted an extensive search of PubMed, the Cochrane Library, Embase, and Web of Science up to December 1, 2024, to identify pertinent randomized controlled trials (RCTs) for inclusion in our study. Our strategy used various keyword combinations related to CS and study design, as detailed in Table S1, Supplemental Digital Content, and was limited to English-language sources.

### 2.2. Eligibility and disqualification methods

The study incorporated all RCTs that evaluated the effectiveness of the atenolol group (AG) compared with a control group (CG), which included sotalol, propafenone, metoprolol, nebivolol, digoxin, or saline, in adults undergoing CS. The main focus was on the occurrence rate of POAF. Secondary outcomes included the necessity for temporary pacemaker insertion due to bradycardia, the rate of dose reduction or drug discontinuation, and mortality. Excluded studies were review articles, abstracts, case reports, or non-English studies. Two investigators (J.H.D. and F.R.H.) independently screened titles and abstracts for eligibility. Ineligible studies were excluded, and the rest underwent full-text analysis to confirm the final inclusion.

### 2.3. Selection of literature and data extraction

Two researchers independently organized the literature with EndNote X9 (Clarivate Analytics)^[19]^ and extracted data on authors, publication year, journal, patient numbers, group distribution, gender, age, surgery type, and outcomes. Discrepancies were resolved through discussion among all authors.

### 2.4. Quality assessment

The evaluation considered factors such as sequence generation, allocation concealment, blinding, data completeness, reporting bias, and other biases, following prescribed guidelines.^[20]^ Methodological rigor was assessed independently with a modified Jadad score,^[21]^ allowing comprehensive evaluation of potential bias and quality in the RCTs.

### 2.5. Statistical analysis techniques

Data analysis used Review Manager 5.3 (The Nordic Cochrane Centre, The Cochrane Collaboration), employing the Mantel–Haenszel method for risk ratios (RRs) and 95% confidence intervals (CIs) for binary outcomes. Subgroup analyses explored the impact of patient characteristics and control interventions. Publication bias was assessed visually with funnel plots. All tests were 2-sided with a significance level of *P* < .05.

### 2.6. Subgroup analysis and heterogeneity

The primary outcomes of this study were POAF and adverse events. POAF was categorized into 3 subgroups based on different control groups: Atenolol and saline, atenolol and sotalol, and atenolol and control (propafenone/metoprolol/nebivolol/digoxin). Postoperative adverse events were further classified into 3 subgroups: temporary pacemaker insertion for bradycardia, rate of dose reduction or drug discontinuation, and mortality.

## 3. Results

### 3.1. Search results

A thorough search of databases such as PubMed, Cochrane Library, Embase, and Web of Science initially identified 230 relevant studies. An extra 65 records were obtained from alternative sources, including reference lists. Nine duplicate studies were removed using EndNote Software (Version X9, Thomson Reuters). Following the screening of titles and abstracts, 116 studies were deemed irrelevant and excluded. After a comprehensive full-text review, an additional 98 studies were excluded. One article could not be obtained in full text. Six studies^[15–17,22–24]^ that satisfied the inclusion criteria were included in this study.

### 3.2. The characteristics of included trials

Table 1 outlines the main features of 6 studies analyzing atenolol’s effectiveness in preventing AF during CS, involving 870 adult patients. These RCTs were conducted between 1988 and 2004, focused on patients undergoing CS. The research was conducted across various countries, including Germany, the United Kingdom, Spain, Belgium, and Turkey. All studies focused on adults undergoing CABG or valve surgery. Atenolol was administered either preoperatively or postoperatively, and the control groups primarily consisted of saline, sotalol, propafenone, metoprolol, nebivolol, and digoxin.

**Table 1:** Characteristics of included trials.

Trials	Sample size	AO clamp time (min) Mean (SD)		Female/male		Protocols		Outcomes
		AG	CG	AG	CG	AG	CG	
Sanjuán 2004^[22]^	253	47 (20)	51 (27)	79/74	32/68	50 mg/d, *po*, pre-op 1d (n = 153)	Sotalol, 80 mg/ bid, *po,* pre-op 1 d (n = 100)	①②③④
Yazicioglu 2002^[15]^	120	57.7 (14.0)	①55.1(14.7); ②60.2(12.7)	8/32	①6/34;②10/30	50 mg/d, *po, pre-op 3 d* (n = 40)	①Digoxin 0.25 mg/d, pre-op 3 d; Extra 1 mg/d, pre-op 2 d, *po* (n = 40); ②Saline (n = 40)	①②③④⑤⑦
Rosada 2000^[16]^	200	NM	NM	10/30	①13/27;②10/30;③13/27;④12/28	25 mg/bid, *po*, post-op 1 d (n = 40)	①Metoprolol, 50 mg/d, bid, *po* (n = 40); ②Sotalol, 80 mg/d, bid, *po* (n = 40); ③Sotalol, 40 mg/d, bid, *po* (n = 40); ④Saline (n = 40)	①②
Merrick 1995^[23]^	207	55.9 (17.9)	52.8 (19.1)	19/83	18/87	50 mg/d, *po*, post-op 7 d (n = 102)	Propafenone, 300 mg/bid, *po*, post-op 7 d (n = 105)	①②③④
Goldstein 1993^[24]^	30	65 (14)	68 (25)	4/11	5/10	50 mg/d, *po*, 10 d after extubation 2 h (n = 15)	Nebivolol, 5 mg/d, *po*, 10 d after extubation 2 h (n = 15)	①⑥
Lamb 1988^[17]^	60	45.1 (14.7)	47.7 (14.0)	3/27	5/25	50 mg/d, *po,* pre-op 3 d and post-op, 7 d (n = 30)	Saline (n = 30)	①⑤

Reported outcomes: ① = Postoperative atrial fibrillation; ② = Temporary pacemaker insertion for bradycardia; ③ = Rate of dose reduction or drug discontinuation; ④ = Mortality; ⑤ = Mean heart rate for the patients with atrial fibrillation; ⑥ = Hemodynamics; ⑦ = Length of stay: ICU stay days, time of extubation (h).

AG = atenolol group, AO = aorta, CG = control group, NM = not mentioned, po *=* peros, post-op = postoperative, pre-op = preoperative, SD = standard deviation.

### 3.3. Potential bias in included studies

**Figure 1.**
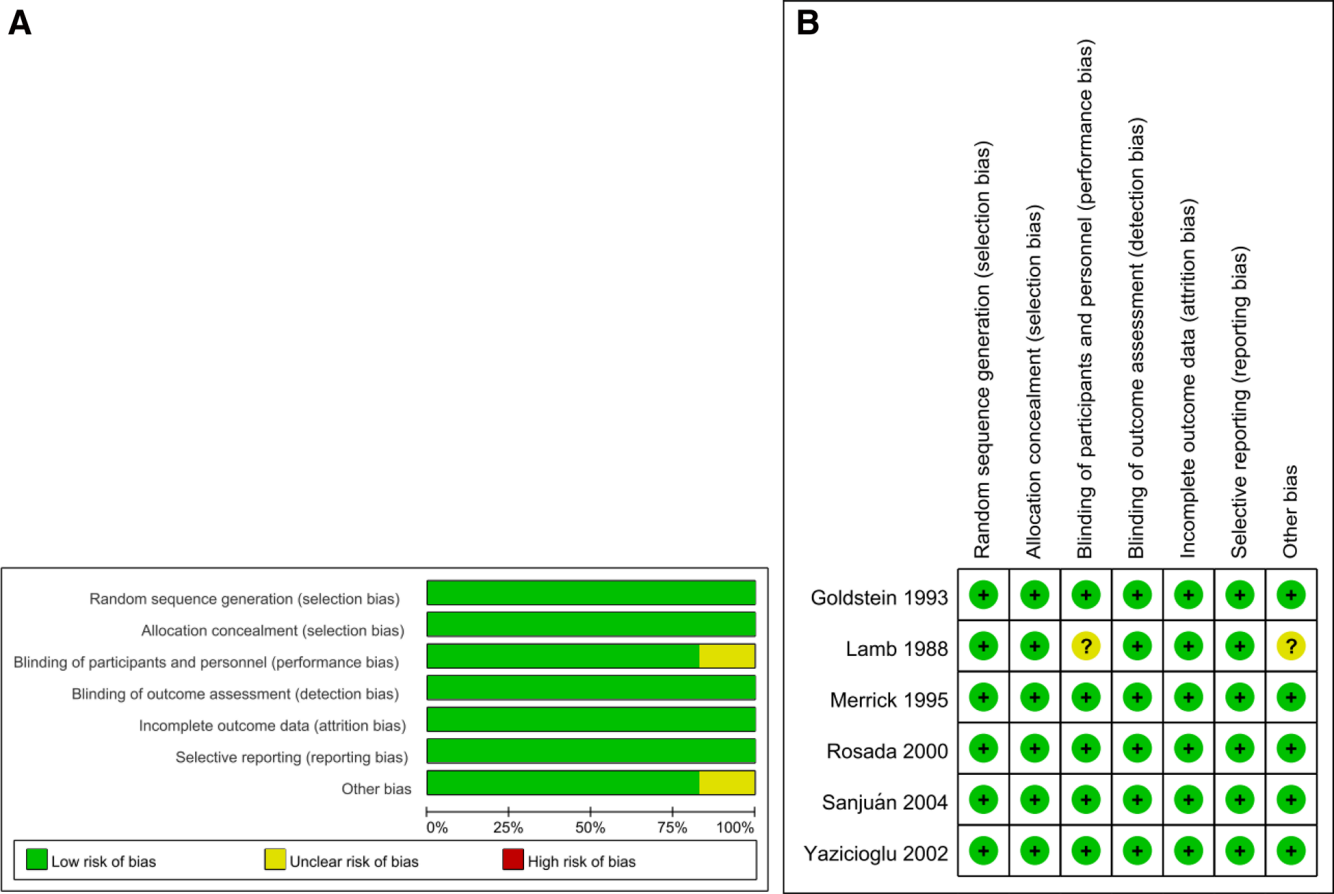
(A) Risk of bias graph for each included study. (B) Risk of bias summary for each included study.

Among the 6 RCTs, 1 lacked details on participant and personnel blinding, indicating an unclear bias risk. In addition, missing details in the table contributed to further bias, complicating the risk assessment (Fig. 1A and B). Among the 6 trials evaluated, 5^[15,16,22–24]^ attained a modified Jadad score of 7, indicating high quality, whereas 1 trial^[17]^ scored 5 (Table 2).

**Table 2:** Modified Jadad score of included trials.

Study	Modified *Jadad* score				
	Randomization	Allocation	Blindness	Withdrawals	Total
Sanjuán 2004^[22]^	2	2	2	1	7
Yazicioglu 2002^[15]^	2	2	2	1	7
Rosada 2000^[16]^	2	2	2	1	7
Merrick 1995^[23]^	2	2	2	1	7
Goldstein 1993^[24]^	2	2	2	1	7
Lamb 1988^[17]^	1	2	2	0	5

### 3.4. The prevention effects on POAF

**Figure 2.**
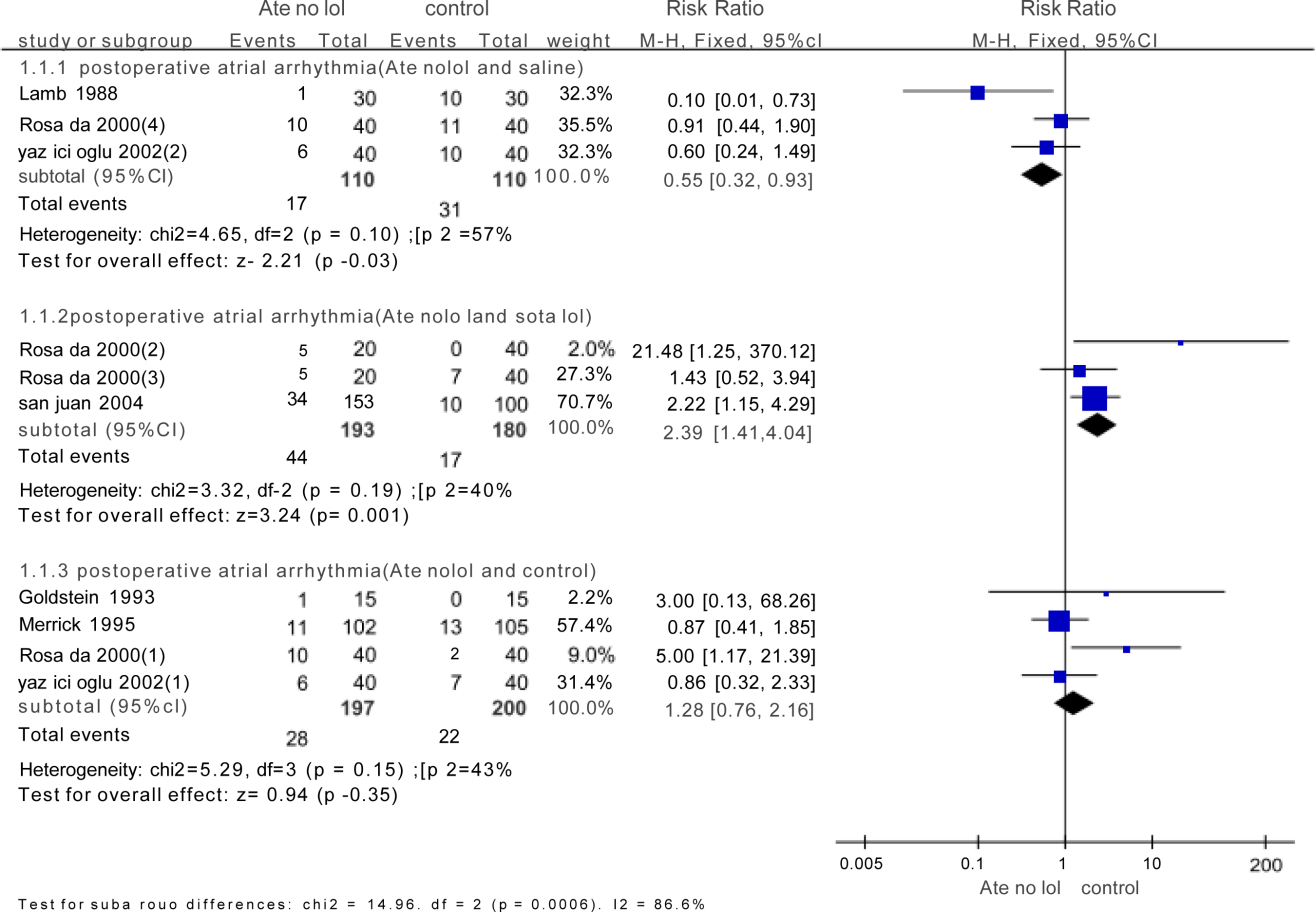
Forest plot comparing atenolol and control for postoperative atrial fibrillation. CI = confidence interval.

The clinical outcomes associated with POAF were analyzed across 6 studies, which encompassed 3 treatment groups. The meta-analysis demonstrated that atenolol significantly lowers the risk of POAF in adults undergoing CS, with an RR of 0.55 (95% CI: 0.32–0.93; *P* = .03) and moderate heterogeneity (*I*^2^ = 57%; *P* = .10). Atenolol was not superior to sotalol in reducing POAF, with an RR of 2.39 (95% CI: 1.41–4.04; *P* = .001) and low heterogeneity (*I^2^* = 40%; *P* = .19). No significant difference in POAF prevention was found between atenolol and the CG (propafenone, metoprolol, nebivolol, and digoxin), with an RR of 1.28 (95% CI: 0.76–2.16; *P* = .35) and moderate heterogeneity (*I*^2^ = 43%; *P *= .15; Fig. 2).

### 3.5. Postoperative adverse events

**Figure 3.**
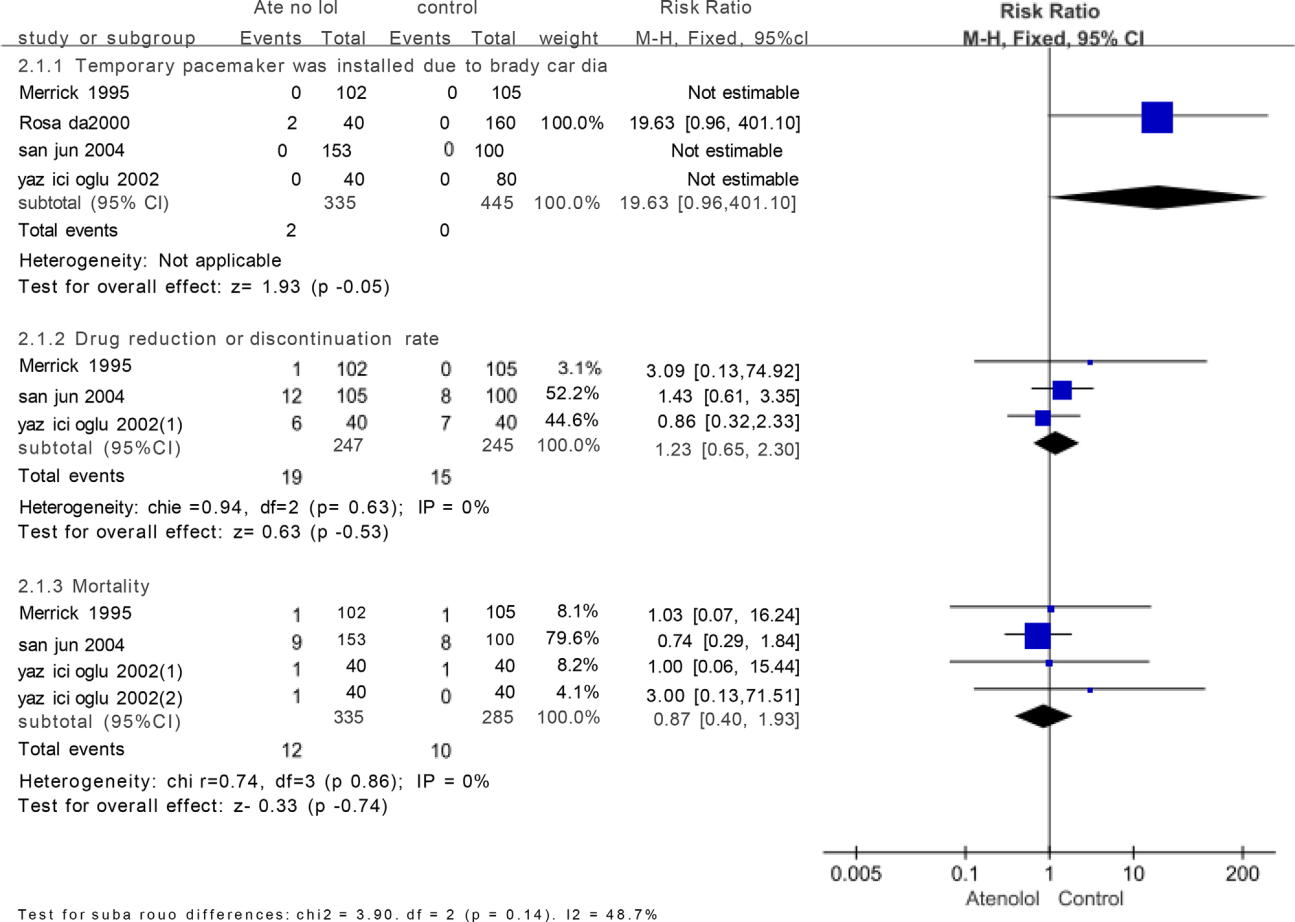
Forest plot comparing atenolol and control for postoperative adverse events. CI = confidence interval.

This study primarily examined the insertion of temporary pacemakers for bradycardia, the rate of dose reduction or drug discontinuation, and mortality, as reported in 4 studies (4 comparisons, 780 patients), 3 studies (3 comparisons, 492 patients), and three studies (3 comparisons, 620 patients), respectively. The study indicated that atenolol did not exert a statistically significant effect on postoperative total adverse events compared to the control (Fig. 3).

### 3.6. Sensitivity testing and publication bias

**Figure 4.**
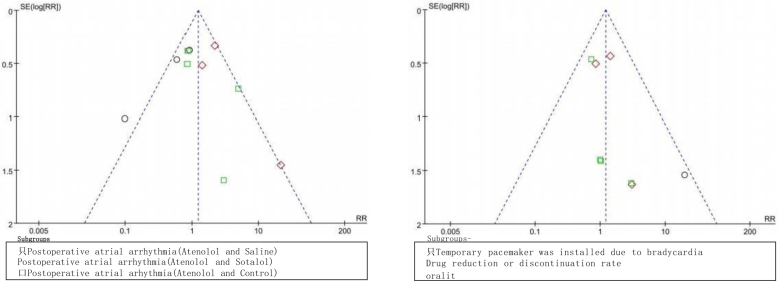
Funnel plot comparing atenolol and control for primary outcomes. RR = risk ratio, SE = standard error.

Sensitivity analysis demonstrated that the results were consistent across different statistical models (Tables 3 and 4). Excluding studies with high heterogeneity did not significantly impact the overall treatment effects (Table 5). It is important to consider the effects of small sample sizes or publication bias. However, funnel plot assessments and Egger’s test (*P* > .05) showed no significant publication bias for the main outcomes (Fig. 4).

**Table 3:** Influence of statistical model on estimated treatment effects of primary outcomes.

Outcomes	Heterogeneity		RR (95% CI)		Overall effect *P*	
	*I* ^2^	*P*	FEM	REM	FEM	REM
Postoperative trial fibrillation (%)						
Atenolol and saline	57	.10	0.55 (0.32–0.93)	0.54 (0.21–1.38)	.03	.20
Atenolol and Sotalol	40	.19	2.39 (1.41–4.04)	2.25 (0.97–5.20)	.001	.06
Atenolol and control	43	.15	1.28 (0.76–2.16)	1.35 (0.59–3.10)	.35	.47
Postoperative adverse events (%)						
Temporary pacemaker insertion for bradycardia	/	/	19.63 (0.96–401.1)	19.63 (0.96–401.1))	.05	.05
Rate of dose reduction or drug discontinuation	0	.63	1.23 (0.65–2.30)	1.20 (0.63–2.26)	.53	.58
Mortality	0	.86	0.87 (0.40–1.93)	0.85 (0.38–1.90)	.74	.69

CI = confidence interval, FEM = fixed effects model, REM = random effects models, RR = risk ratio.

**Table 4:** Sensitivity analyses of the influence of individual study on the overall effects.

Outcomes	Excluded trials	Heterogeneity		RR		95% CI		Overall effect *P*	
		*I*^2^ (%)	*P*	FEM	REM	FEM	REM	FEM	REM
Postoperative trial fibrillation (Atenolol and Sotalol)	Rosada 2000 (2)	0	.47	2.00	1.95	1.15–3.48	(1.12–3.39)	.01	.02
Postoperative trial fibrillation (Atenolol and control)	Rosada 2000 (1)	0	.75	0.92	0.91	0.51–1.65	(0.50–1.64)	.78	.74

CI = confidence interval, FEM = fixed effects model, REM = random effects models, RR = risk ratio.

**Table 5:** Sensitivity analyses of high heterogeneity outcomes.

Heterogeneity outcomes	Excluded trials	Heterogeneity		RR		95% CI		Overall effect *P*	
		*I*^2^ (%)	*P*	FEM	REM	FEM	REM	FEM	REM
Postoperative trial fibrillation (Atenolol and saline)	Lamb 1988	0	.49	0.76	0.77	0.43–1.35	(0.44–1.37)	.35	.38

CI = confidence interval, FEM = fixed effects model, REM = random effects models, RR = risk ratio.

## 4. Discussion

This meta-analysis is the first to assess atenolol’s effectiveness and safety in preventing POAF in CS patients, indicating its potential to reduce this condition. POAF, a common postsurgery complication, predicts adverse outcomes and may highlight patients at higher risk for complications, stroke, and mortality.^[2,10,11]^ A study of 6475 CABG patients found that 15% developed AF, with higher mortality rates both in-hospital (7.4% vs 3.4%) and 4 years postsurgery (26% vs 13%). In a matched cohort, 5-year mortality was higher for AF patients (20% vs 7%).^[3]^ POAF is also linked to increased all-cause mortality at 30 days and 6 months postsurgery.^[3^^,25–31]^ Importantly, AF should not be regarded solely as an early postoperative complication; it is also associated with adverse long-term outcomes, including increased mortality in selected patient populations. Notably, the prognostic significance of AF extends beyond the CS setting. In elderly patients without heart failure undergoing hip fracture surgery, preoperative AF has been identified as an independent predictor of long-term mortality,^[32]^ underscoring that AF serves as a marker of heightened cardiovascular risk with implications for long-term survival. These findings further strengthen the rationale for early risk stratification and support a more personalized approach to POAF prevention, as identifying high-risk individuals may enable interventions that improve both short- and long-term clinical outcomes.

This meta-analysis showed that atenolol significantly lowers the incidence of POAF in patients undergoing CS when used as a prophylactic intervention. When comparing the AG with the saline group, the prevalence of POAF was 15.5% versus 28.2% (*P* = .03).^[15–17]^ Several studies have compared the AG with the sotalol group, indicating that atenolol is less effective than sotalol in preventing POAF, with prevalence rates of 22.8% versus 9.4% (*P* = .001).^[16,22]^ In contrast, when compared with the CG, which included propafenone, metoprolol, nebivolol, and digoxin, the AG exhibited similar efficacy in preventing POAF after CS (14.2% vs 11.0%; *P* = .35).^[15,16,23,24]^ The administration of beta-blockers remains the most widely employed prophylactic strategy, supported by numerous studies highlighting their benefits, ease of use, and cost-effectiveness.^[1,2,33–36]^ The findings suggest that initiating beta-blocker therapy either preoperatively or immediately postoperatively can reduce the risk of AF. The available evidence does not support recommending 1 beta-blocker over another. These recommendations are consistent with guidelines from the American College of Cardiology Foundation/American Heart Association, Society of Thoracic Surgeons, American Association of Thoracic Surgery, Canadian Cardiology Society, and the European Society of Cardiology.^[37–39]^ This meta-analysis indicates that atenolol significantly lowers the incidence of POAF in CS compared with a placebo. Its efficacy is comparable with that of the control group (propafenone/metoprolol/nebivolol/digoxin), yet it is notably less effective than sotalol. It is important to highlight that this study encompasses a limited number of studies, suggesting that additional high-quality RCTs are necessary for further validation.

The precise etiological factors and mechanisms underlying the development of POAF remain elusive, encompassing aspects such as oxidative stress, sympathetic nervous system activation, and acute inflammation. Moreover, individuals with a history of cardiac conditions may exhibit structural abnormalities, ion channel dysfunctions, and alterations in atrial architecture.^[40,41]^ The study indicates that oxidative stress following cardiac tissue reperfusion in CABG patients may increase nicotinamide adenine dinucleotide phosphate oxidase activity in atrial appendage tissue. Elevated oxidase activity is a notable independent predictor for the development of POAF.^[42]^

Atenolol is associated with several common clinical side effects, including sinus bradycardia and hypotension. Notably, the 6 studies incorporated in our analysis did not reveal any significant differences in postoperative side effects between the AG and the CG.^[15–17,22–24]^ Our meta-analysis indicated that the AG exhibited a marginally higher incidence of temporary pacemaker insertion due to bradycardia compared with the CG (0.6% vs 0%; *P* = .05). The AG and CG showed similar results for dose reduction or drug discontinuation rates (7.69% vs 6.12%; *P *= .53) and mortality (3.58% vs 3.51%; *P* = .74). Despite low heterogeneity, the limited number of studies in the analysis requires cautious interpretation of the findings.

## 5. Limitations

Our study is subject to several limitations. First, the analysis is limited by a small sample size, as it includes only 6 studies, and relies on outdated articles. Second, the study focused solely on English-language publications, potentially leading to publication bias. Third, no studies in our analysis monitored patients long term.

## 6. Conclusions

Recent studies suggest that atenolol could be a safe and effective intervention for the prevention of POAF in adult patients undergoing CS.

## Author contributions

**Formal analysis:** Jin-He Deng.

**Investigation:** Jin-He Deng.

**Writing – original draft:** Jin-He Deng.

**Data curation:** Fan-Rong He.

**Methodology:** Fan-Rong He.

**Software:** Fan-Rong He.

**Conceptualization:** Yun-Tai Yao.

**Supervision:** Yun-Tai Yao.

**Writing – review & editing:** Yun-Tai Yao.


