# Supplementary material for: A meta-analysis of randomized controlled trials on atenolol’s impact on postoperative atrial fibrillation in adults undergoing cardiac surgery
Source: Medicine (Baltimore). 2026 Jul 31;105(31):e50029. doi: 10.1097/MD.0000000000050029 (PMC13433016; doi:10.1097/MD.0000000000050029)
Supplement: Supplementary file 1 [file medi-105-e50029-s001.doc]

**Supplement Table 1.** Database search strategies.

| Search strategies | Results |
| --- | --- |
| **Pubmed**  ((((((atenolol) AND (cardiac surgery)) AND (randomized controlled trial)) OR (((atenolol) AND (cardiopulmonary bypass) AND (randomized controlled trial)))) OR (((atenolol) AND (coronary artery bypass grafting) AND (randomized controlled trial)))) OR (((atenolol) AND (valve surgery) AND (randomized controlled trial)))) OR (((atenolol) AND (aortic surgery) AND (randomized controlled trial))) | 4/54 |
| **Cochrane**  ((((((atenolol) AND (cardiac surgery)) AND (randomized controlled trial)) OR (((atenolol) AND (cardiopulmonary bypass) AND (randomized controlled trial)))) OR (((atenolol) AND (coronary artery bypass grafting) AND (randomized controlled trial)))) OR (((atenolol) AND (valve surgery) AND (randomized controlled trial)))) OR (((atenolol) AND (aortic surgery) AND (randomized controlled trial))) in Title Abstract Keyword | 0/21 |
| **Embase**  (('atenolol'/exp OR atenolol) AND ('cardiac surgery'/exp OR 'cardiac surgery' OR (('cardiac'/exp OR cardiac) AND ('surgery'/exp OR surgery))) AND ('randomized controlled trial'/exp OR 'randomized controlled trial' OR (randomized AND controlled AND ('trial'/exp OR trial))) OR (('atenolol'/exp OR atenolol) AND ('cardiopulmonary bypass'/exp OR 'cardiopulmonary bypass' OR (cardiopulmonary AND ('bypass'/exp OR bypass))) AND ('randomized controlled trial'/exp OR 'randomized controlled trial' OR (randomized AND controlled AND ('trial'/exp OR trial)))) OR (('atenolol'/exp OR atenolol) AND ('coronary artery bypass grafting'/exp OR 'coronary artery bypass grafting' OR (coronary AND ('artery'/exp OR artery) AND ('bypass'/exp OR bypass) AND ('grafting'/exp OR grafting))) AND ('randomized controlled trial'/exp OR 'randomized controlled trial' OR (randomized AND controlled AND ('trial'/exp OR trial)))) OR (('atenolol'/exp OR atenolol) AND ('valve surgery' OR (('valve'/exp OR valve) AND ('surgery'/exp OR surgery))) AND ('randomized controlled trial'/exp OR 'randomized controlled trial' OR (randomized AND controlled AND ('trial'/exp OR trial)))) OR (('atenolol'/exp OR atenolol) AND ('aortic surgery'/exp OR 'aortic surgery' OR (aortic AND ('surgery'/exp OR surgery))) AND ('randomized controlled trial'/exp OR 'randomized controlled trial' OR (randomized AND controlled AND ('trial'/exp OR trial))))) AND  ('randomized controlled trial'/de) | 2/130 |
| [**Web of Science**](http://webofscience--clarivate--cn--https.clarivate.gzzyy.yuntsg.cn:2222/wos/)  TS=(((((((atenolol) AND (cardiac surgery)) AND (randomized controlled trial)) OR (((atenolol) AND (cardiopulmonary bypass) AND (randomized controlled trial)))) OR (((atenolol) AND (coronary artery bypass grafting) AND (randomized controlled trial)))) OR (((atenolol) AND (valve surgery) AND (randomized controlled trial)))) OR (((atenolol) AND (aortic surgery) AND (randomized controlled trial)))) and Preprint Citation Index (Exclude – Database) and Clinical Trial (Document Types) | 0/25 |
